# Polo-Like Kinase Controls Vertebrate Spindle Elongation and Cytokinesis

**DOI:** 10.1371/journal.pone.0000409

**Published:** 2007-05-02

**Authors:** Ian M. Brennan, Ulf Peters, Tarun M. Kapoor, Aaron F. Straight

**Affiliations:** 1 Department of Biochemistry, Stanford Medical School, Stanford, California, United States of America; 2 Laboratory of Chemistry and Cell Biology, Rockefeller University, New York, New York, United States of America; Fred Hutchinson Cancer Research Center, United States of America

## Abstract

During cell division, chromosome segregation must be coordinated with cell cleavage so that cytokinesis occurs after chromosomes have been safely distributed to each spindle pole. Polo-like kinase 1 (Plk1) is an essential kinase that regulates spindle assembly, mitotic entry and chromosome segregation, but because of its many mitotic roles it has been difficult to specifically study its post-anaphase functions. Here we use small molecule inhibitors to block Plk1 activity at anaphase onset, and demonstrate that Plk1 controls both spindle elongation and cytokinesis. Plk1 inhibition did not affect anaphase A chromosome to pole movement, but blocked anaphase B spindle elongation. Plk1-inhibited cells failed to assemble a contractile ring and contract the cleavage furrow due to a defect in Rho and Rho-GEF localization to the division site. Our results demonstrate that Plk1 coordinates chromosome segregation with cytokinesis through its dual control of anaphase B and contractile ring assembly.

## Introduction

The process of mitosis distributes chromosomes into two new daughter cells. The mitotic spindle controls both the movement of chromosomes in mitosis and the division of cells in cytokinesis. During anaphase, chromosomes are separated by moving from the metaphase plate to the spindle pole (anaphase A) and by the elongation of the mitotic spindle (anaphase B). In cytokinesis, the position of the mitotic spindle directs the assembly and contraction of an actomyosin ring, midway between the spindle poles, to cleave the cell. Although the mitotic spindle directs both the segregation of chromosomes and the specification of the cleavage plane, the mechanisms that initiate anaphase spindle dynamics and that communicate spindle position to the site of contractile ring formation are not known [1], [2].

Chromosome segregation, spindle dynamics and cytokinesis must be tightly coordinated to ensure proper cell division. A key factor in regulating transitions through mitosis is the polo like kinase 1, Plk1. Plk1 function has been implicated in centrosome maturation, mitotic spindle assembly, cyclin dependent kinase activation, kinetochore function, chromosome cohesion, mitotic exit and cytokinesis (reviewed in [3]). However, analyzing the specific role of Plk1 during anaphase and cytokinesis has been particularly difficult because inhibition of Plk1 activity by siRNA or genetic mutation causes defects early in mitosis [4].

Anaphase chromosome to pole movement is triggered by dissolving the link between sister chromatids. Artificially separating sister chromatids is sufficient for anaphase A [5], thus chromosome to pole movement appears to result from a change in the balance between sister chromatid cohesion and forces pulling chromosomes toward the spindle pole. In budding yeast, sister chromatid cohesion also regulates anaphase B as loss of chromosome cohesion is sufficient to trigger spindle elongation [6]. The regulation of metazoan spindle elongation is more complex. Removal of all chromosomes from the spindle does not result in anaphase spindle elongation [7] thus there must exist a trigger other than chromosome cohesion to initiate spindle elongation. Although the exact mechanism for anaphase B is unknown, Plk1 localizes to the spindle midzone immediately after anaphase, is known to directly phosphorylate the midzone kinesin MKLP2 [8] and is required for the midzone localization of the MKLP1 kinesin [9]. Thus Plk1 is a candidate for controlling anaphase spindle elongation but its role in the process has not been defined.

Contractile ring assembly begins immediately after anaphase chromosome segregation and requires the contractile ring localization of the small GTPase Rho. Blocking Rho activity inhibits cleavage furrow assembly and ingression [10]–[13]. Rho regulates cytokinesis through activation of specific Rho effector molecules that stimulate actin polymerization at the contractile ring, that regulate the contractile activity of nonmuscle myosin II, and that promote cell abscission (reviewed in [14]).

Rho activity is regulated by the Rho guanine nucleotide exchange factor ECT2 (hereafter Rho-GEF) and the Rho GTPase activating protein complex, centralspindlin, composed of the mitotic kinesin MKLP1 and the Rho GAP MgcRacGAP (hereafter Rho-GAP) [15]–[18]. Rho-GAP localizes to the microtubules of the spindle midzone and the tips of astral microtubules through its kinesin subunit [19]–[21], Rho-GEF binds Rho-GAP and localizes to the midzone and cell cortex [20], [22], [23] and Rho localizes to the cell cortex at the site of furrow formation [10], [24]. Disruption of either Rho-GEF or Rho-GAP activity causes Rho mislocalization and cytokinesis failure [20], [22], [23], [25], [26].

The budding yeast polo kinase (Cdc5) has recently been demonstrated to regulate Rho activity in cytokinesis by phosphorylation of Rho-GEF. Two Rho-GEF proteins (Tus1 and Rom2) are Cdc5 substrates and mutation of Cdc5 or the Rho-GEF proteins disrupted Rho activation at the bud neck and blocked contractile actin ring formation [27]. Budding yeast cytokinesis is unique in that cleavage plane specification is not microtubule dependent and occurs at bud emergence rather than at the time of cytokinesis [28] thus it is an interesting question as to whether Plk1 controls vertebrate cytokinesis in an analogous manner. Human cells expressing kinase dead Plk1 failed to complete cytokinesis [29] and expression of nondegradable Plk1 mutants in HeLa cells delayed cleavage furrow contraction and reduced the cleavage furrow ingression rate [30]. Depletion of Plk1 using siRNA results in a prometaphase arrest and the formation of monopolar spindles [4], [31]. The mitotic arrest caused by Plk1 depletion has made it difficult to study cytokinesis in Plk1 depleted cells, however it has been reported that cells depleted of Plk1 do not show a defect in the initiation or progression of cytokinesis [31].

The recent development the Plk1 chemical inhibitors DAP81, BTO-1/cyclapolin1 and BI-2536 has enabled the study of Plk1 in cytokinesis [32]–[34]. Because these molecules can diffuse into cells and rapidly inactivate Plk1 they provide an opportunity to study the role of Plk1 in animal cell cytokinesis without the secondary effects that result from Plk1 depletion by RNAi.

We used small molecule inhibitors of Plk1 to block Plk1 activity at the metaphase to anaphase transition. Rapid inhibition of Plk1 blocked spindle elongation and cytokinesis. Anaphase B is completely absent in Plk1 inhibited cells but anaphase A chromosome to pole movement is unaffected. We show that Plk1 inhibition blocks contractile ring assembly by preventing the localization of Rho and Rho-GEF without affecting the assembly of the central spindle. Our data suggests that Plk1 controls anaphase spindle elongation and contractile ring assembly to coordinate chromosome segregation with cytokinesis in animal cells.

## Results

### Polo kinase activity is essential for cytokinesis

**Figure 1:**
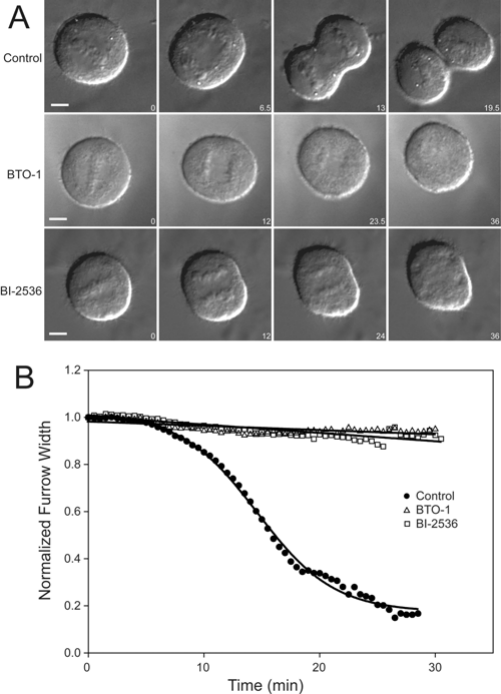
Plk1 inhibition prevents cytokinesis. A) DIC images taken from timelapse recordings of HeLa cells progressing through cytokinesis in the presence or absence of Plk1 inhibitors. Untreated cells are shown in the top row and cells treated with either BTO-1 or BI-2536 are shown in the middle and bottom rows, respectively. Time (minutes) after anaphase onset is shown in the bottom right corner of each image. B) Degree of furrow contraction in the presence or absence of Plk1 inhibitors. The measured width of the furrow is normalized to the pre-anaphase width. The half-time of furrow ingression for the control cells is 15.32±2.77 minutes. No ingression was observed in cells treated with BTO-1 or BI-2536. T_1/2_ is shown±SD. Each curve represents the average of multiple timelapse recordings (Control n = 10, BTO-1 n = 10, and BI-2536 n = 10).

We investigated the role of Plk1 during cytokinesis by testing the effects of two different Plk1 inhibitors, BTO-1 and BI-2536, on cells initiating cytokinesis. To avoid perturbing spindle assembly or chromosome segregation, we monitored HeLa cells by differential interference contrast (DIC) microscopy, added inhibitor just as chromosomes separated at anaphase and then monitored the progression of cytokinesis. Treatment of HeLa cells with either BTO-1 or BI-2536 completely blocked the ingression of the cleavage furrow resulting in cytokinesis failure (Figure 1A, Video S1, S2 and S3). Untreated HeLa cells showed significant contraction within 15 minutes of anaphase onset (Figure 1A, Control 13 minutes, Figure 1B Control) and completed most of furrow contraction within 20 minutes (Figure 1A, Control 19.5 minutes, Figure 1B Control). Plk1 inhibited cells had not contracted 25 minutes after anaphase onset (Figure 1A, BTO-1 23.5 minutes and BI-2536 24 minutes, Figure 1B BTO-1 and BI-2536) and eventually began to decondense chromosomes without cell cleavage (Figure 1A, BTO-1 36 minutes and BI-2536 36 minutes, Figure 1B BTO-1 and BI-2536). Plk1 inhibition also blocked cytokinesis in the kangaroo rat PtK_2_ cell line (Figure S1, Video S4 and S5). In the more adherent PtK_2_ cell line, we observed some furrow contraction late in cytokinesis but furrow contraction was significantly reduced in rate and extent (Figure S1, Video S5). This is consistent with the previous observation that more adherent cell lines can contract in the absence of active Rho but that less adherent HeLa cells cannot [13]. The use of two structurally unrelated Plk1 inhibitors in two different cell types suggests that the effects we observe are specific to Plk1 inhibition. Our observed inhibition of contractile ring ingression by BTO-1 and BI-2536 indicates a role for Plk1 activity early in cytokinesis during the specification or assembly phase of the contractile ring or a mechanical block to furrow ingression as seen with myosin II inhibition [35].

### Anaphase B spindle elongation requires polo kinase activity

**Figure 2:**
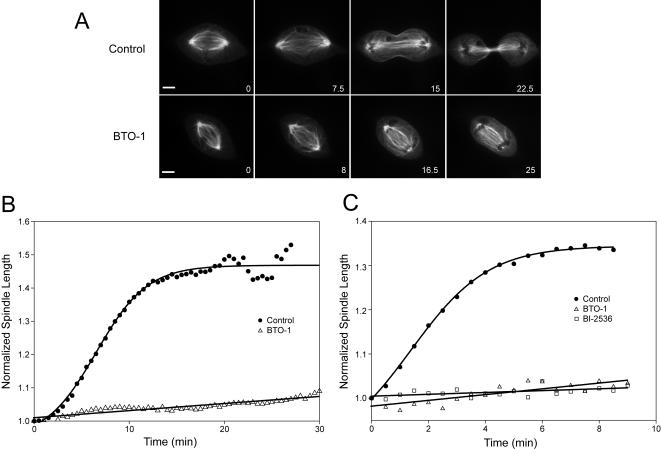
Plk1 inhibition blocks anaphase B spindle elongation. A) Fluorescence images taken from timelapse recordings of GFP-Tubulin expressing PtK2 cells in the presence or absence of Plk1 inhibitor. Time shown in lower right had corner represents minutes after anaphase onset. Untreated cells are shown in the top row and BTO-1 treated cells are shown in the bottom row. B) Degree of anaphase B inhibition in PtK_2_ cells treated with BTO-1. Spindle length is normalized to the length of the pre-anaphase spindle. The half-time of spindle elongation for untreated PtK_2_ cells is 6.57±0.58 minutes. None of the cells treated with BTO-1 achieved greater than a 10% increase in spindle length. Curves represent an average of Control (n = 22) and BTO-1 (n = 10) recordings. C) Degree of anaphase B inhibition in HeLa cells treated with BTO-1 or BI-2536. Graphs are as described in B (Control n = 10, BTO-1 n = 10, BI-2536 n = 10). The half-time of spindle elongation untreated HeLa cells is 2.31±0.66 minutes. No spindle pole separation was observed in Plk1 inhibited cells. All measurements are shown±SD.

We examined the effect of Plk1 inhibition on the microtubule spindle to determine whether spindle disruption might be the cause of our observed cytokinesis defect. Using timelapse fluorescence microscopy, we monitored anaphase spindle dynamics in PtK_2_ cells stably expressing GFP-α−Tubulin [36]. Treatment of cells with Plk1 inhibitor did not affect the formation of the spindle midzone and anaphase A chromosome to spindle pole movement occurred at the same rate as in untreated cells (Figure S2, Video S6). However, drug treatment caused a block in the elongation of the spindle during anaphase B (Video S7, Figure 2A and 2B). This effect was also apparent in HeLa cells treated with BTO-1 or with BI-2536 (Figure 2C). Quantitation of the anaphase B defects in PtK_2_ cells (Figure 2B) and HeLa cells (Figure 2C) showed that no spindle elongation occurred during the time period when spindle elongation completed in control cells.

### The competence windows for anaphase B and cytokinesis are distinct

**Figure 3:**
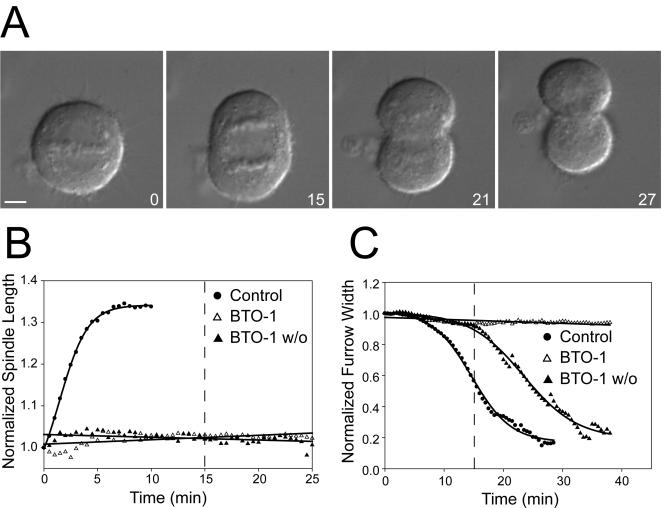
Reversibility of Plk1 inhibition of cytokinesis and anaphase B. A) DIC images from a timelapse video of HeLa cells treated with BTO-1 for 15 minutes after anaphase onset. Inhibitor was removed at 15 minutes, second panel represents first timelapse image after inhibitor removal. Time in minutes after anaphase is shown in the lower right hand corner. B) Normalized spindle length in cells after inhibitor washout. Control data is the same data shown previously in Figure 2C for reference. BTO-1 washout (closed triangles) represents an average of 5 timelapse recordings. C) Normalized furrow width showing cytokinesis recovery after BTO-1 washout (closed triangles) (n = 5). Dashed lines represent time of washout. The half-time of furrow ingression following BTO-1 washout is 23.46±3.72 minutes post anaphase onset. T_1/2_ is shown±SD.

We examined the reversibility of Plk1 inhibition by removing the drug and following cytokinesis and anaphase B recovery by timelapse microscopy. We treated HeLa cells with BTO-1 for 15 minutes after anaphase onset, a time at which most of anaphase B and greater than 50 percent of cleavage furrow contraction are complete in untreated cells, and then removed the drug by exchange with fresh culture medium. HeLa cells rapidly resumed furrow contraction after BTO-1 washout (Figure 3A and 3C, Video S8) but anaphase B spindle elongation did not recover (Figure 3A and 3B, Video S8). The competence window for cytokinesis referred to as C-phase lasts for 60–90 minutes after anaphase onset [37], [38]. Our data suggests that there is a more restricted window for anaphase B execution than for cytokinesis. Previous observations demonstrated that actin or myosin inhibition during cytokinesis does not affect anaphase B [35], [39], [40]. Our results imply that spindle elongation and cytokinesis are separable events that are independently controlled. We were unable to wash out BI-2536 and get recovery of cytokinesis. We suspect that this reflects a difference in affinity between BTO-1 and BI-2536 for Plk1. The IC50 for Plk1 inhibition by BTO-1 is 8 µM and the IC50 for BI-2536 is 800 pM thus there may not be adequate time for BI-2536 dissociation and cytokinesis recovery in our experiments.

### Plk inhibition blocks contractile ring assembly

**Figure 4:**
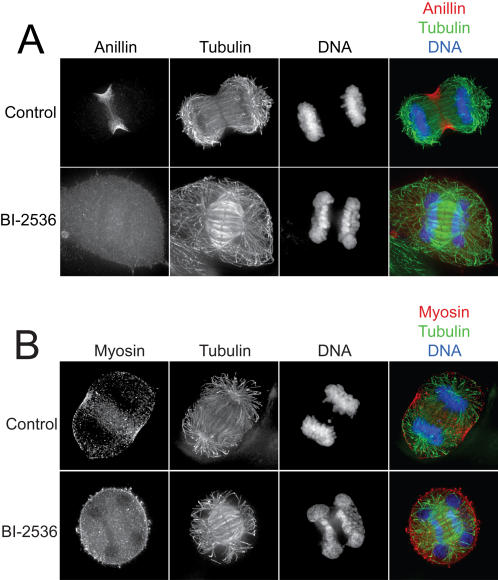
Plk1 inhibition prevents cleavage furrow assembly. A) Fluorescence images of control or BI-2536 treated HeLa cells. Top row shows localization of anillin, tubulin and DNA in untreated cells, bottom row shows the same in BI-2536 treated cells. B) Same as depicted in A but localization of myosin II is shown. Scale bar represents 5 µm.

The assembly of the actomyosin contractile ring is dependent upon Rho effector molecules. We previously showed that kinase inhibitors that disrupted the spindle midzone or blocked ROCK activity resulted in a loss of myosin II from the contractile ring but did not affect the localization of the contractile ring protein anillin [35]. Anillin requires Rho for its contractile ring localization but does not depend on any of known Rho effectors for its contractile ring assembly [17], [41], [42]. We examined the localization of anillin and myosin II after Plk1 inhibition and found that both anillin and myosin II were delocalized from the contractile ring (Figure 4A, 4B). This suggests that Plk1 is acting early in the contractile ring assembly pathway to block both anillin and myosin II localization and thus the primary effect of Plk1 inhibition is likely on the Rho pathway.

### Polo kinase is required for Rho localization during cytokinesis

**Figure 5:**
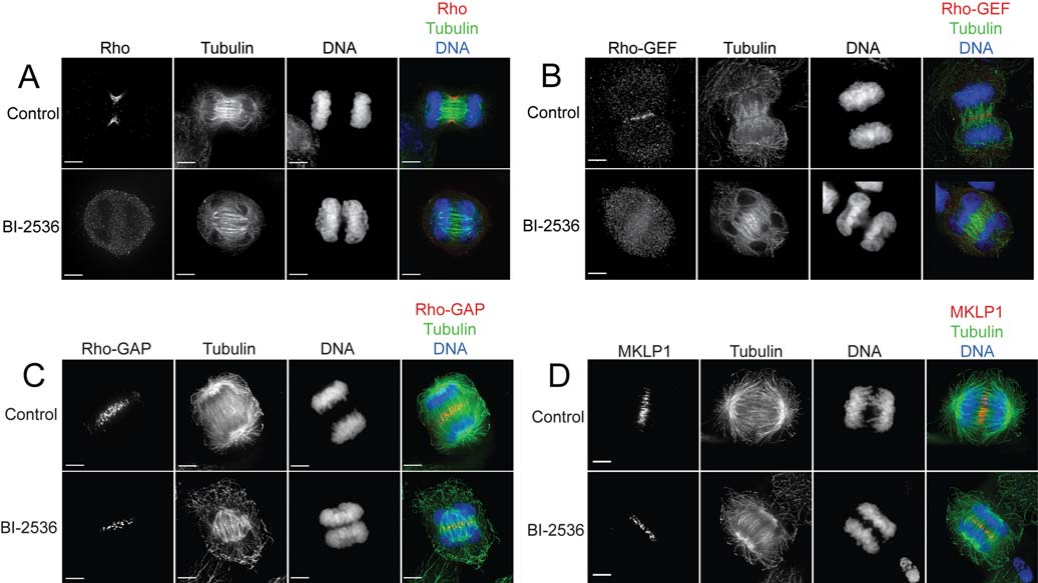
Plk1 inhibition blocks the localization of Rho and Rho-GEF but not Rho-GAP or MKLP1. A) Fluorescence images of control or BI-2536 treated HeLa cells. Top row shows localization of Rho, Tubulin and DNA in untreated cells and bottom row shows localization after BI-2536 treatment. B) Localization of Rho-GEF as described for A. C) Localization of Rho-GAP (MgcRacGAP) as described for A. D) Localization of MKLP1 as described for A. Scale bar represents 5 µm.

We investigated the mechanism of Plk1 inhibition of cytokinesis by examining the localization Rho and its regulators after Plk1 inhibition. HeLa cells treated with BI-2536 or BTO-1 failed to accumulate Rho and Rho-GEF at the contractile ring (Figure 5A, 5B). In untreated mid-anaphase cells, 94±3% of cells showed proper Rho localization and 96±0% showed Rho-GEF localization while only 2±3% of BI-2536 treated cells localized Rho and 2±3% of cells localized Rho-GEF. The centralspindlin complex consisting of Rho-GAP and MKLP1 was still able to localize in Plk1 inhibited cells suggesting that Plk1 activity is not required for Rho-GAP localization (Figure 5C, 5D). Plk1 no longer localized to the central spindle in the presence of BI-2536 (Figure S3). These results suggest that Plk1 could be acting at the midzone to control the localization of Rho or Rho-GEF or the activity of Rho-GAP or Rho-GEF.

## Discussion

Accurate cell division requires coordinating spindle dynamics, chromosome segregation and cell cleavage and Plk1 integrates these diverse processes. Here we demonstrate two novel functions for Plk1 in vertebrates. Plk1 is essential for anaphase spindle elongation and Plk1 initiates cytokinesis by controlling Rho localization to the contractile ring. Our observations demonstrate that Plk1 controls the switch from metaphase anaphase spindle dynamics and that Plk1 governs the timing of contractile ring assembly and thus may function in the signaling pathway from microtubules that specifies the cleavage plane.

Chromosomes are segregated by chromosome to pole movement and spindle elongation in anaphase. Anaphase B spindle elongation involves motor proteins acting at the center of the spindle to push the spindle poles away from one another, motor proteins acting on astral microtubules to pull spindle poles toward the cell cortex and changes in microtubule dynamics [43] but the mechanisms that coordinate these activities are not known. Although metaphase spindle length increases when kinetochore-microtubule interactions are weakened [44] sister chromatid cohesion appears to primarily resist chromosome to pole movement and not spindle elongation.

Our observation that Plk1-inhibited cells perform anaphase A normally but are blocked in anaphase B suggests that a simple force-balance mechanism through kinetochore microtubule attachment does not govern anaphase B in vertebrate mitosis. Instead, Plk1 activity at anaphase onset is necessary for spindle elongation to occur. Plk1 phosphorylation could either activate motor proteins to drive antiparallel microtubule sliding at anaphase or could release a rigor state so that the forces that separate spindle poles were no longer resisted by crosslinking of midzone microtubules. Plk1 may also alter the dynamics of microtubules as microtubule polymerization and midzone stabilization is necessary for efficient anaphase B.

Microtubule motor proteins have been implicated in anaphase spindle elongation and thus are potential targets for Plk1 regulation. Kinesin-5 motors that crosslink and slide antiparallel microtubules contribute to bipolar spindle formation and spindle pole separation in several organisms [45]–[48]. Members of the kinesin-4 and kinesin-6 families localize to the central spindle in anaphase (Kurasawa et al., 2004; Kwon et al., 2004; Williams et al., 1995, Nislow et al., 1992) and cytoplasmic dynein contributes to spindle pole separation by sliding astral microtubules along the cell cortex [49], [50]. Thus it will be interesting to determine whether Plk1 regulates of anaphase B through changes in motor protein activity.

Our studies demonstrate that Plk1 is essential for the assembly of the contractile ring and the initiation of cytokinesis. Plk1 depletion by siRNA results in several early mitotic defects that have hampered studying the cytokinesis specific functions of Plk1 [4], [31]. By using fast acting chemical inhibitors we have circumvented the problems associated with siRNA depletion of Plk1 and demonstrated that Plk1 activity at anaphase onset is essential for cytokinesis. Our observations on the role of Plk1 in cytokinesis are consistent with studies of human cells engineered to express an ATP analog sensitive allele of Plk1 [51].

Plk1 inhibition blocked the signaling pathways that initiate contractile ring formation. Plk1 is required for Rho and Rho-GEF to localize properly but does not control Rho-GAP/MKLP1 localization to the central spindle. Plk1 is also localized to the central spindle during anaphase implicating Rho-GAP and Rho-GEF as potential targets of Plk1 phosphorylation. The Rho-GAP/MKLP1 complex binds Rho-GEF and is essential for its localization, thus one role for Plk1 might be to regulate the interaction between Rho-GEF and Rho-GAP by direct phosphorylation of either protein [20], [22], [23], [26].

In budding yeast, the Rho-GEF Tus1 is phosphorylated by Cdc5. Replacement of the Cdc5 phsophorylation sites with phosphomimetic mutants partially bypassed the requirement for Cdc5 in Rho localization and actin ring formation whereas mutation of the phosphorylation sites to nonphosphorylatable residues blocked Rho localization and actin assembly. Cytokinesis in yeast and vertebrates differs in that yeast myosin II localizes to the bud neck in Cdc5 mutants [27], whereas Rho inhibition in vertebrate cells leads to myosin II delocalization [13]. It will be interesting to determine whether Plk1 is controlling contractile ring formation by regulating the interactions or activities of Rho-GAP and Rho-GEF in vertebrate cells.

Small molecule inhibitors of Plk1 have allowed us to determine the specific contribution of Plk1 to anaphase and cytokinesis in vertebrate cells. Our discovery that Plk1 is required to initiate anaphase B will help to dissect the mechanisms that control the switch from metaphase to anaphase spindle dynamics required for chromosome segregation. Our observation that Plk1 activity is required to localize Rho to the contractile ring is an important step toward understanding how the microtubule spindle communicates its position to the cell cortex to specify the cleavage plane and initiate contractile ring assembly. Understanding how Plk1 controls anaphase and cytokinesis will require identifying the relevant Plk1 targets that affect Rho localization and spindle elongation. The dual role for Plk1 in controlling anaphase B initiation and cytokinesis suggests that Plk1 is central to the coordination of chromosome segregation in anaphase and cell cleavage.

## Materials and Methods

### Cell Culture and Inhibitor Treatment

HeLa cells and HeLa cells stably expressing GFP-centrin were cultured in DMEM (GIBCO) supplemented with 10% fetal bovine serum (FBS) at 37°C in 5% CO_2_. PtK_2_ cells expressing GFP-Tubulin were cultured in Kaighn's Modification of Ham's F12 (GIBCO) supplemented with 10% fetal bovine serum (FBS) at 37°C in 5% CO_2_. BTO-1 was used at 20 µM and BI-2536 was used at 250 nM. Inhibitors were diluted from DMSO stock solutions into warm media and used immediately.

### Inhibitor Synthesis

BTO-1 [33] and BI-2536 [52] were synthesized as previously described.

### Antibodies

DM1A anti-tubulin antibody was purchased from Sigma and used at 1 µg/ml. Antibodies to MKLP1, Rho, ECT2 (Santa Cruz Biotechnology) and human nonmuscle myosin II (BTI) were used at 1 µg/ml. MgcRacGAP antibody was provided by Wei-Meng Zhao and Guowei Fang and used at 0.5 µg/ml. Anillin antibody was a gift from Christine M. Field and used at 1 µg/ml.

### Fixation and Immunofluorescence of HeLa cells

HeLa cells were treated with inhibitor for 30 minutes on coverslips and then fixed for immunofluorescent staining. Cells were fixed with 4% formaldehyde in 60 mM piperazine-*N,N'*-bis(2-ethanesulfonic acid), 25 mM HEPES, 0.2% Triton-X100 10 mM EGTA, 4 mM MgSO_4_ at pH 7.0 for 10 minutes at 37°C to localize anillin, myosin II, RhoGAP (MgcRacGAP), Mklp1, and Mklp2. Cell fixation was quenched with Tris buffered saline (TBS) containing 0.2% Triton-X100 and 30mM glycine for 5 minutes at room temperature. To localize RhoGEF, cells were fixed in 100% methanol at −20°C for 1 minute and then immediately rehydrated in TBS. Localization of Rho was performed after fixation in ice cold 10% trichloroacetic acid (TCA) for 10 minutes as previously described [20]. Fixed cells were blocked in antibody dilution buffer (TBS, 0.1% Triton-X100, 0.1% sodium azide, 2% bovine serum albumin) and all subsequent staining was performed in the same buffer. Secondary antibodies were purchased from Molecular Probes or Jackson Immunoresearch and used as recommended. DNA was stained by incubation with 1 µg/ml Hoechst (Sigma) in antibody dilution buffer for 10 minutes at room temperature. Cells were mounted in 90% glycerol containing 0.5% p-phenylenediamine at pH8.8.

### Microscopy

Timelapse microscopy was performed on a Nikon TE2000 inverted microscope with a 100×1.4NA PlanApo objective. Images were collected through a Yokogawa CSU-10 spinning disc confocal scanner. Image data was acquired with a Cascade 512B CCD camera (Photometrics) using Metamorph software for device control and data collection. For imaging experiments, cells were transferred to Leibovitz L-15 without phenol red, and supplemented with 10% FBS. Cells were maintained at 37°C on the microscope stage using a custom designed stage incubator. Fixed cell immunofluorescence images were acquired using an Olympus IX-70 inverted microscope with a 100×1.4NA PlanApo objective. Three-dimensional data was collected by axial sectioning with an automated stage and images were acquired and deconvolved using DeltaVision SoftWorx software (Applied Precision). All fluorescence images represent maximum intensity projections in two dimensions of three dimensional image data.

### Data Analysis

Timelapse data was analyzed to calculate contractile ring ingression and spindle elongation. Contractile ring ingression was calculated by measuring the distance from one side of the furrow to the other across the cell in DIC images using Metamorph software. The pre-anaphase distance was calculated by measuring the cell diameter across the metaphase plate. The distance values were divided by the pre-anaphase cell width to give a normalized value so that multiple datasets could be compared and averaged. Anaphase B data was calculated using GFP-Tubulin images (Ptk_2_ cells) or GFP-centrin images (HeLa cells) or DIC images (HeLa cells) by measuring the length of the spindle between the two spindle poles. Data was normalized as for cytokinesis but using the pre-anaphase spindle length as the reference point. The data were fit to least squares regression lines using SigmaPlot software (SysStat). Half times and maximum or average velocities for furrow ingression and spindle pole separation were calculated from the regression lines with Mathematica software (Wolfram Research).

## Supporting Information

Figure S1 Plk1 inhibition prevents cytokinesis in PtK2 cells. A) DIC images taken from timelapse recordings of PtK2 cells progressing through cytokinesis in the presence or absence of Plk1 inhibitors. Untreated cells are shown in the top row and cells treated with BTO-1 are shown in the bottom row. Time (minutes) after anaphase onset is shown in the bottom right corner of each image. B) Degree of furrow contraction in the presence or absence of Plk1 inhibitors. The measured width of the furrow is normalized to the pre-anaphase width. Each curve represents the average of multiple timelapse recordings (Control n = 22, BTO-1 n = 10).
(5.22 MB TIF)

Figure S2 Anaphase A is not affected by Plk1 inhibition. Chromosome to pole distance was measured for 16 chromosomes in 8 separate timelapse recordings for both control and BTO-1 inhibited cells. The average velocity calculated in the linear range between 0 and 4 minutes is 0.87±0.19 µm/min in control cells and 0.76±0.14 µm/min in BTO-1 treated cells.
(1.16 MB TIF)

Figure S3 Plk1 inhibition blocks Plk1 localization A) Fluorescence images of control or BI-2536 treated HeLa cells. Top row shows localization of Plk1, tubulin and DNA in untreated cells, bottom row shows the same in BI-2536 treated cells. Scale bar represents 5 µm.
(4.53 MB TIF)

Video S1 DIC timelapse of untreated HeLa cell mitosis.
(0.89 MB MOV)

Video S2 DIC timelapse of BTO-1 treated HeLa cell mitosis.
(1.02 MB MOV)

Video S3 DIC timelapse of BI-2536 treated HeLa cell mitosis.
(0.71 MB MOV)

Video S4 DIC timelapse of untreated PtK2 cell mitosis.
(2.39 MB MOV)

Video S5 DIC timelapse of BTO-1 treated PtK2 cell mitosis.
(1.92 MB MOV)

Video S6 Simultaneous fluorescence and DIC timelapse of GFP-tubulin expressing PtK2 cell mitosis.
(2.96 MB MOV)

Video S7 Simultaneous fluorescence and DIC timelapse of BTO-1 treated, GFP-tubulin expressing, PtK2 cell mitosis.
(3.46 MB MOV)

Video S8 DIC timelapse of BTO-1 treated HeLa cell with drug washout.
(0.88 MB MOV)
