# Supplementary figures and images for: Polo-Like Kinase Controls Vertebrate Spindle Elongation and Cytokinesis
Source: PLoS One. 2007 May 2;2(5):e409. doi: 10.1371/journal.pone.0000409 (PMC1853238; doi:10.1371/journal.pone.0000409)

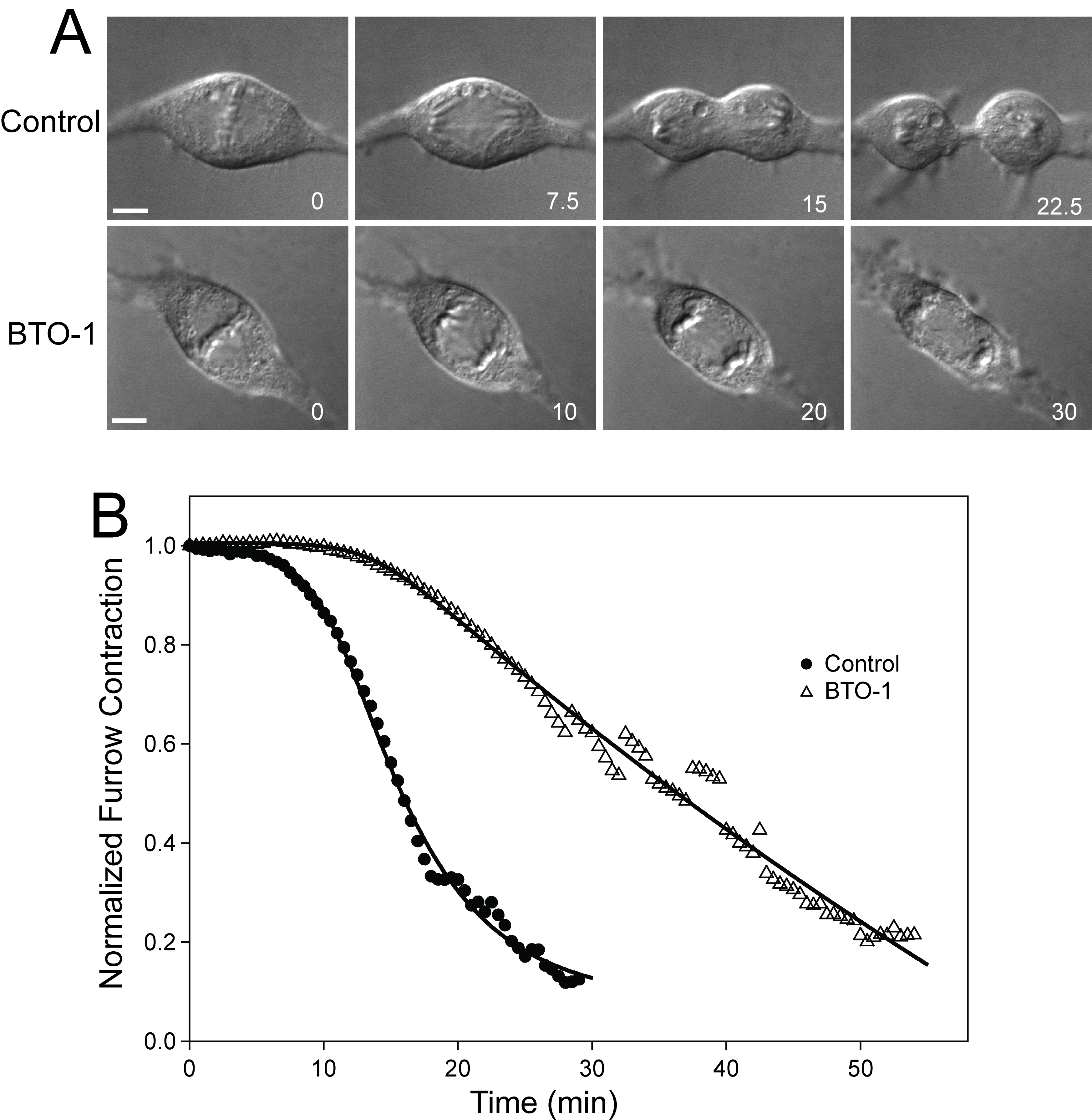

Supplement: Figure S1 — Plk1 inhibition prevents cytokinesis in PtK2 cells. A) DIC images taken from timelapse recordings of PtK2 cells progressing through cytokinesis in the presence or absence of Plk1 inhibitors. Untreated cells are shown in the top row and cells treated with BTO-1 are shown in the bottom row. Time (minutes) after anaphase onset is shown in the bottom right corner of each image. B) Degree of furrow contraction in the presence or absence of Plk1 inhibitors. The measured width of the furrow is normalized to the pre-anaphase width. Each curve represents the average of multiple timelapse recordings (Control n = 22, BTO-1 n = 10). (5.22 MB TIF) [file pone.0000409.s001.tif]

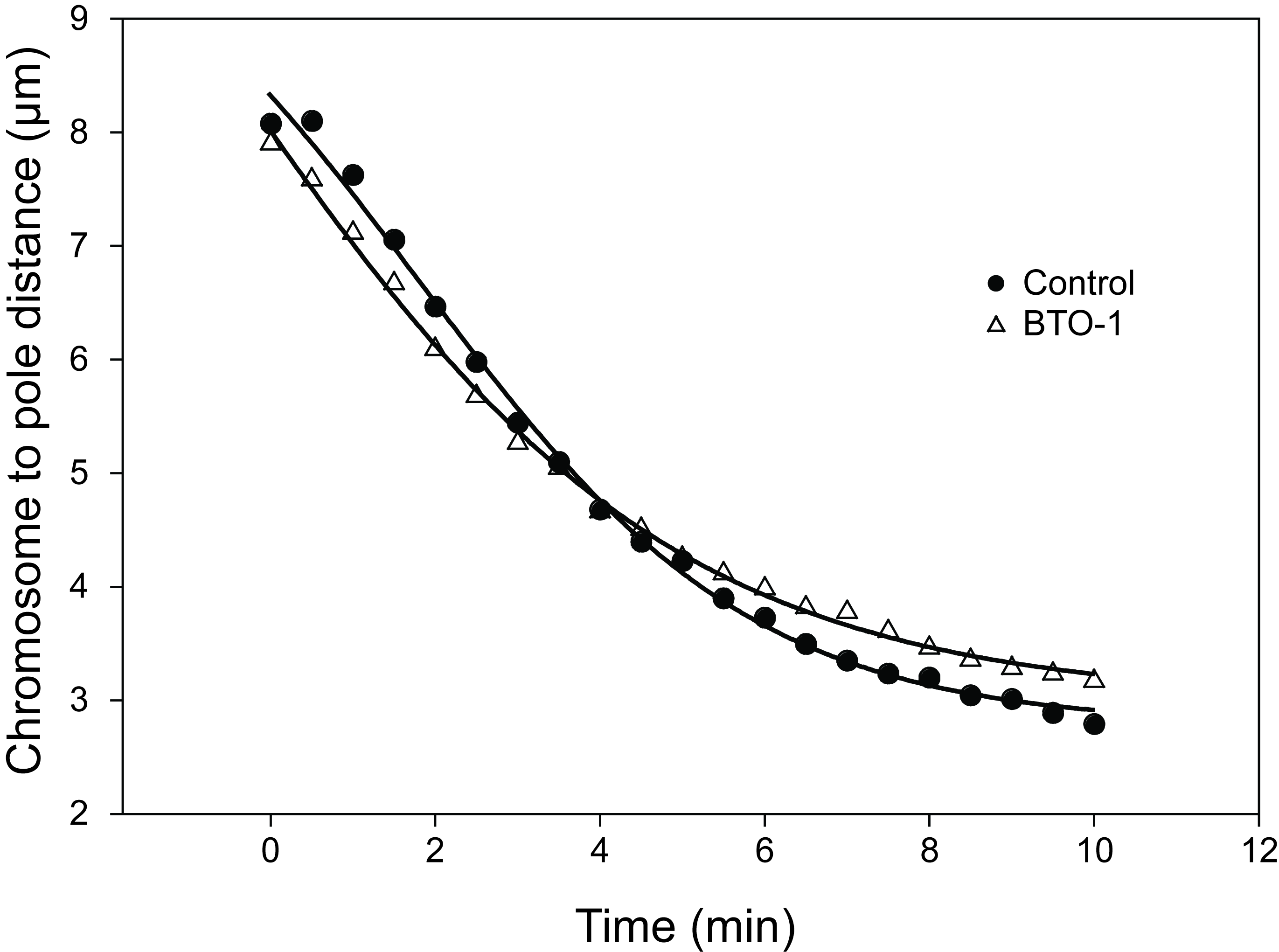

Supplement: Figure S2 — Anaphase A is not affected by Plk1 inhibition. Chromosome to pole distance was measured for 16 chromosomes in 8 separate timelapse recordings for both control and BTO-1 inhibited cells. The average velocity calculated in the linear range between 0 and 4 minutes is 0.87±0.19 µm/min in control cells and 0.76±0.14 µm/min in BTO-1 treated cells. (1.16 MB TIF) [file pone.0000409.s002.tif]

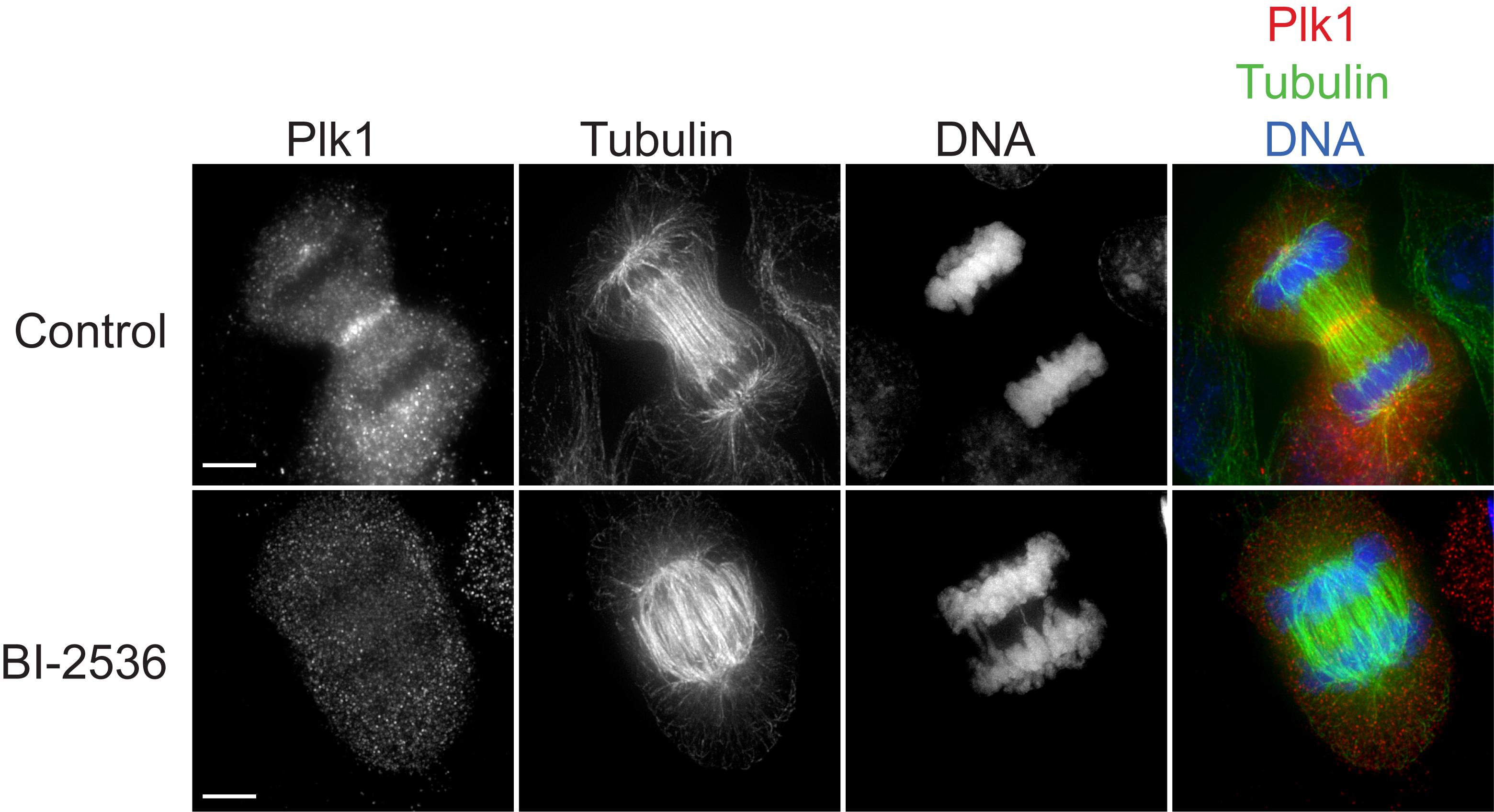

Supplement: Figure S3 — Plk1 inhibition blocks Plk1 localization A) Fluorescence images of control or BI-2536 treated HeLa cells. Top row shows localization of Plk1, tubulin and DNA in untreated cells, bottom row shows the same in BI-2536 treated cells. Scale bar represents 5 µm. (4.53 MB TIF) [file pone.0000409.s003.tif]
